# A Theoretical Approach to the Effect of Substrate Engineering on Hydrogen–Metal Surface Coverage in Ir–Pt Nanocatalysts for Water Electrolysis

**DOI:** 10.1021/acsomega.6c05827

**Published:** 2026-08-21

**Authors:** Farid Taherkhani, Fabian Mauss

**Affiliations:** † 38871 Departments of Thermodynamics and Thermal Process Engineering Brandenburg University of Technology, Cottbus 03046, Germany; ‡ Energiespeicher- Und Energiewandlersysteme, Brandenburgische Technische Universität Cottbus−Senftenberg, Cottbus 03046, Germany

## Abstract

A novel analytical expression for the surface coverage of metal hydrogen has been derived as a function of the Gibbs free energy of the Tafel step, electrical overpotential, chemical reaction rate constant, and activation energy of the Tafel step for the hydrogen evolution reaction (HER) in alkaline media. Analytical solutions for the surface coverage of metal–hydrogen have been obtained in both the low- and high-temperature limits in terms of the same key parameters. An asymptotic limit of surface coverage as a function of applied overpotential is observed, even at elevated temperatures under alkaline conditions. The HER performance of Ir–Pt nanoalloys supported on different substrates has also been studied in alkaline media. The sensitivity of metal–hydrogen surface coverage to kinetic parameters on three different substrate materials CNTs, Ti sheet, and stainless steel for the Ir–Pt nanocatalyst has been analyzed with respect to overpotential for the HER in water electrolysis in alkaline media by a theoretical approach. The computational results indicate that the sensitivity of surface coverage is closely related to the water dissociation reaction on the catalytic surface, specifically the formation of adsorbed hydrogen on the metal surface and the concurrent release of hydroxide ions. The exchange current density of Ir–Pt nanoalloys in the hydrogen evolution reaction (HER) during water electrolysis in acidic media has been systematically investigated via DFT calculations. The DFT calculation results show that doping Pt with Ir to form Ir–Pt nanoalloys makes a significant contribution to the exchange current density. The DFT results show that the exchange current density of Ir–Pt nanoalloys exhibits a nonmonotonic dependence on Ir content. The calculated DFT exchange current densities for Ir, Pt, and Ir–Pt nanoalloys are in good agreement with available experimental data.

## Graphical abstract

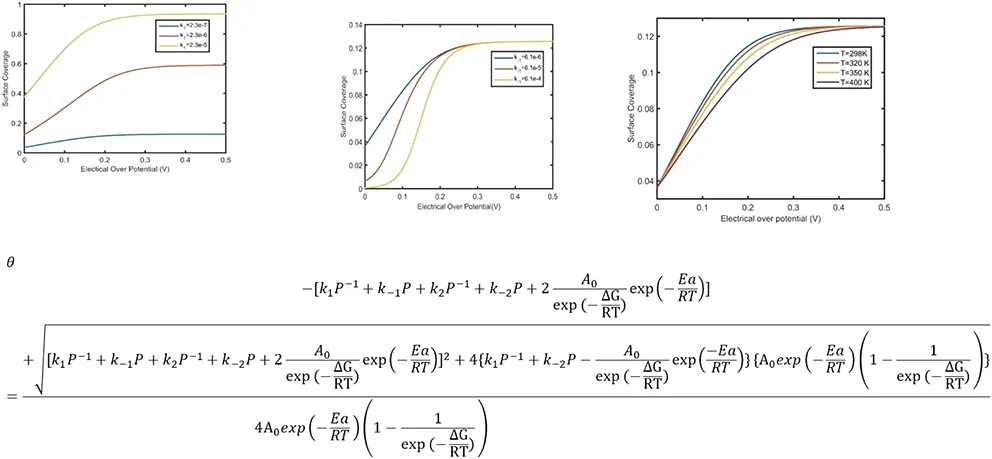

## 1 Introduction

The hydrogen evolution reaction (HER) is a fundamental electrochemical process for sustainable hydrogen production through water electrolysis and is considered to be a cornerstone technology for future clean energy systems. In alkaline media, the HER has attracted significant attention because alkaline electrolyzers offer advantages such as lower operational costs, enhanced durability, and compatibility with non-noble-metal catalysts. However, HER kinetics in alkaline environments are generally slower than in acidic media due to the additional water-dissociation step required to generate adsorbed hydrogen intermediates (H*). Therefore, the development of highly efficient and stable electrocatalysts for the alkaline HER remains a major scientific challenge.^1,2^

Nanostructured electrocatalysts have emerged as promising candidates for improving the HER performance because of their large surface area, tunable electronic properties, and high density of catalytically active sites. Transition-metal-based nanomaterials, including alloys, phosphides, sulfides, nitrides, and carbides, have been extensively investigated as cost-effective alternatives to platinum-based catalysts.^3,4^ In recent years, increasing attention has been devoted to understanding the role of catalyst–support interactions and substrate effects in determining electrocatalytic activity.

The substrate plays a critical role not only as a mechanical support for catalyst growth but also as an active component that influences the catalytic process. Strong electronic interactions between the nanocatalyst and the substrate can induce charge redistribution, modify the surface electronic structure, and optimize the adsorption/desorption energies of hydrogen intermediates.^5^ In the alkaline HER, where water dissociation is often the rate-determining step, substrate-induced modulation of interfacial electronic properties can significantly accelerate reaction kinetics.^6^

Furthermore, conductive substrates facilitate efficient electron transfer and improve catalyst stability during long-term electrolysis. Substrates such as nickel foam, carbon cloth, graphene, titanium mesh, and copper foam have been widely employed due to their high conductivity, porous structure, and large electrochemically active surface area.^7^ Three-dimensional porous substrates are particularly advantageous because they enhance electrolyte penetration, gas diffusion, and bubble release, thereby improving the mass transport and catalytic efficiency.

In addition to electronic effects, substrate morphology and surface chemistry strongly influence catalyst nucleation, dispersion, adhesion, and durability. Interfacial strain effects and synergistic interactions between nanocatalysts and substrates can generate new active sites and improve the intrinsic catalytic activity.^8^ Consequently, substrate engineering has become an essential strategy for designing next-generation HER electrocatalysts with enhanced performance and long-term operational stability.

A comprehensive understanding of substrate effects in alkaline HER is therefore crucial for advancing efficient hydrogen production technologies. Optimizing catalyst–substrate interfaces through rational materials design offers significant opportunities to improve electrocatalytic activity, reduce overpotential, and enhance durability in practical alkaline water electrolysis systems.

The HER has been extensively studied on different metal surfaces using density functional theory (DFT) calculations,^9^ and the exchange current density has been evaluated for the HER on various metal surfaces in acidic media.^10^ Mixed Ni–Co-oxide cathodes with different compositions have also been fabricated via thermal decomposition and applied as electrocatalysts for hydrogen production in both acidic and alkaline media. These oxide electrodes generally exhibit semicrystalline structures, contributing to their catalytic activity.^9^

Furthermore, Pt nanowire-based electrodes have shown a significant enhancement in current density during water electrolysis in acidic media.^11^ Cobalt nanoparticles encapsulated in nitrogen-doped carbon (Co@N–C) have also demonstrated high activity and durability for HER across a wide pH range, as well as bifunctional activity for the oxygen evolution reaction (OER) in alkaline media.^12^

Kinetics modeling for water electrolysis in alkaline media has been investigated using Raney nickel electrodes (Ni–Al–Cu–Cr alloy) through AC impedance and DC polarization current measurements in a 5.36 M KOH solution at 70 °C.^13^ Similarly, HER kinetics at Pd–Ni electrodeposited alloy electrodes has been studied using AC impedance spectra and DC polarization in a 0.5 M NaOH solution at room temperature.^14^ The mechanism of HER on modified Raney nickel composite-coated electrodes has also been analyzed via AC impedance for kinetic modeling in alkaline media.^15^

The relationship between electrical circuit parameters and excess overpotential has been explored across different electrode materials, redox couples, and supporting electrolytes.^16^ Impedance spectroscopy modeling has been further applied to electrochemical systems containing heterocharge redox couples in the presence of supporting electrolytes.^17^ DFT calculations have emerged as an essential tool for estimating the electrical current density in electrochemical systems with fabricated electrodes made from diverse materials.^18,19^

Electrodes fabricated with nanoparticle-based materials have been shown to significantly enhance current density for the HER in both acidic and alkaline media.^20^ Moreover, Co-based selenide electrodes in phosphate buffer demonstrate excellent performance for saline water electrolysis.^21^

From these studies, several open questions remain, particularly regarding the derivation of a closed analytical form for metal–hydrogen surface coverage as a function of electrical overpotential, the Gibbs free energy of the Tafel step, the activation energy of the Tafel step, and the behavior of metal–hydrogen surface coverage at low and high temperatures in alkaline media. In alkaline media, the effect of the substrate on the sensitivity of the metal–hydrogen surface coverage during the hydrogen evolution reaction (HER) requires further investigation across all reaction steps in the presence of Ir–Pt nanocatalysts. Additionally, the role of thermal energy in controlling the surface coverage of adsorbed hydrogen on metal surfaces during HER in alkaline water electrolysis, as well as its temperature dependence, remains unclear. Furthermore, the exchange current density for HER in acidic media on electrodes fabricated from Pt, Ir, and Ir–Pt nanoalloys still requires comprehensive investigation.

## 2 Theoretical Methods

### 2.1 Hydrogen Evolution Reaction in Acidic Media

HER during water electrolysis in acidic media on a metal surface electrode can be summarized as the reactions in eqs 1–3.

1 $$M+H^{+}+e⇌\mathrm{MH}\left(\mathrm{ad}\right),\mathrm{Volmer}$$

2 $$\mathrm{MH}\left(\mathrm{ad}\right)+H^{+}+e⇌H_{2}+M,\mathrm{Heyrovsky}$$

3 $$\mathrm{MH}\left(\mathrm{ad}\right)+\mathrm{MH}\left(\mathrm{ad}\right)⇌H_{2}+2M,\mathrm{Tafel Step}$$

A schematic presentation of the mechanism of HER in water electrolysis in acidic media is presented in Figure 1 which described Volmer, Heyrovsky, and Tafel step.

**Figure 1:**
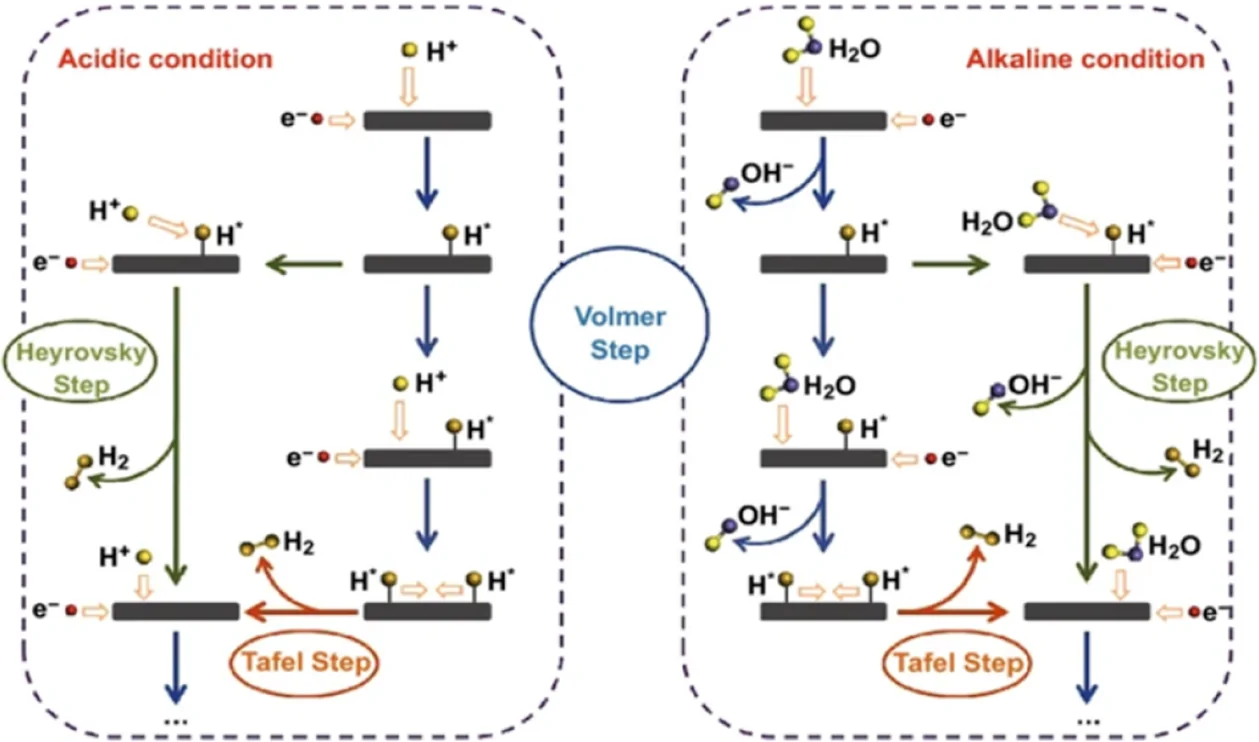
Schematics presentation for HER in water electrolysis on a catalytic surface in acidic and alkaline media.

### 2.2 Hydrogen Evolution Reaction in Alkaline Media

The HER for water electrolysis in alkaline media can be imagined as eqs 4–6.

4 $$M+H_{2}O+e⇌\mathrm{MH}\left(\mathrm{ad}\right){+\mathrm{OH}}^{-}$$

5 $$\mathrm{MH}\left(\mathrm{ad}\right)+H_{2}O+e⇌H_{2}+M+{\mathrm{OH}}^{-}$$

6 $$\mathrm{MH}\left(\mathrm{ads}\right)+\mathrm{MH}\left(\mathrm{ads}\right)⇌H_{2}+2M$$

Schematics representation for the HER in water electrolysis in alkaline media which is described based on eqs 4–6 is presented in Figure 1.

### 2.3 Dependence of Hydrogen Adsorption Surface Coverage in Surface Catalysis on Temperature, Activation Energy, and Gibbs Free Energy

We obtain the surface coverage θ which shows H adsorbing on the catalytic surface, versus the electrical overpotential η. Electrochemical rate equations for the forward reactions 4–5, *K*_1_ and *K*_2_, could be written based on the activation energy barrier (*B* × *F* × η) by using an Arrhenius like equation.^20,21^

7 $$K_{1}=k_{1}\;\exp\left(\left(-B\times F\times \eta \right)/\left(R\times T\right)\right)$$

8 $$K_{2}=k_{2}\;\exp\left(\left(-B\times F\times \eta \right)/\left(R\times T\right)\right)$$

In eqs 4–6 backward chemical rate reaction *K*_–1_ and *K*_–2_ can be written as^20,21^

9 $$K_{-1}=k_{-1}\exp\;\left(\left(1-B\right)\times F\times \eta /\left(R\times T\right)\right)$$

10 $$K_{-2}=k_{-2}\exp\;\left(\left(1-B\times F\times \eta \right)/\left(R\times T\right)\right)$$

In eqs 7–10, *k*_1_, *k*_2_, *k*_3_, *k*_–1_, *k*_–2_, and *k*_–3_ are electron rate constant for forward and backward of electrochemical reactions 4–6, respectively.

The rate of surface coverage, θ, can be written as

11 $$r_{1}=k_{1}\left(1-\theta \right)\exp\left(-\beta \frac{F\eta }{RT}\right)-k_{-1}\theta \exp\left(\frac{\left(1-\beta \right)F\eta }{RT}\right)$$

12 $$r_{2}=k_{2}\theta \exp\left(-\beta \frac{F\eta }{RT}\right)-k_{-2}\left(1-\theta \right)\exp\left(\frac{\left(1-\beta \right)F\eta }{RT}\right)$$

13 $$r_{3}=k_{3}\theta^{2}-k_{-3}{\left(1-\theta \right)}^{2}$$

H* (hydrogen on the metal surface) is a short-lived intermediate. It is continuously generated and consumed. The concentration of H* did not accumulate indefinitely. As a result, the steady state approximation has been assumed to get the surface coverage as a function of time.

We use the steady state condition for surface coverage:

14 $$r_{1}+r_{2}-2r_{3}=0$$

The chemical reaction in eq 6 is not an electrochemical reaction with cooperative electron transfer. It is just a chemical reaction. Then we use Arrhenius equation for the chemical reaction in eq 6:

15 $$k_{3}=A_{0}\;\exp\left(-\frac{Ea}{RT}\right)$$

*Ea*, *T*, and *A*_0_ are the activation energy barrier in the Tafel step, temperature, and prefactor, respectively.

The backward rate of the chemical reaction can be related to the Gibbs free energy (ΔG = −*RT* ln *K*) of the Tafel step reaction eq 6 and the forward reaction as following:

16 $$K=\frac{k_{3}}{k_{-3}},,k_{-3}=\frac{k_{3}}{\exp\left(-\frac{\Delta G}{\mathrm{RT}}\right)}$$

17 $$k_{-3}=\frac{k_{3}}{K}=\frac{A_{0}}{\exp\left(-\frac{\Delta G}{\mathrm{RT}}\right)}\exp\left(-\frac{\mathrm{Ea}}{RT}\right)$$

By definition, the following variable

18 $$C=k_{3}-k_{-3}=A_{0}\exp\left(-\frac{Ea}{RT}\right)\left(1-\frac{1}{\exp\left(-\frac{\Delta G}{\mathrm{RT}}\right)}\right)$$

19 $$S=k_{1}P^{-1}+k_{-1}P+k_{2}P^{-1}+k_{-2}P+2\frac{A_{0}}{\exp\left(-\frac{\Delta G}{\mathrm{RT}}\right)}\exp\left(-\frac{Ea}{RT}\right)$$

20 $$B^{\prime }=k_{1}P^{-1}+k_{-2}P-\frac{A_{0}}{\exp\left(-\frac{\Delta G}{\mathrm{RT}}\right)}\exp\left(\frac{-Ea}{RT}\right)$$

where

21 $$P=\exp\left(\frac{\beta F\eta }{RT}\right),,P^{-1}=\exp\left(-\frac{\beta F\eta }{RT}\right)$$

Surface coverage could be written as following:

22 $$\theta =\frac{-S+\sqrt{S^{2}+4B^{\prime }C}}{4C}$$

By substituting *S*, *B*′, and *C* from eqs 18–20 in eq 22, we get an explicit form of the surface coverage of hydrogen on the catalytic surface based on activation energy and Gibbs free energy in the Tafel step, with temperature and electrical overpotential as variables, as eq 23:

23 $$\theta =\frac{\begin{array}{l} -\left[k_{1}P^{-1}+k_{-1}P+k_{2}P^{-1}+k_{-2}P+2\frac{A_{0}}{\exp\left(-\frac{\Delta G}{RT}\right)}\exp\left(-\frac{Ea}{RT}\right)\right]\\ +\sqrt{{[k_{1}P^{-1}+k_{-1}P+k_{2}P^{-1}+k_{-2}P+2\frac{A_{0}}{\exp\left(-\frac{\Delta G}{RT}\right)}\exp\left(-\frac{Ea}{RT}\right)]}^{2}+4{\{k}_{1}P^{-1}+k_{-2}P-\frac{A_{0}}{\exp\left(-\frac{\Delta G}{RT}\right)}\exp\left(\frac{-Ea}{RT}\right)\}\left\{A_{0}\exp\left(-\frac{Ea}{RT}\right)\left(1-\frac{1}{\exp\left(-\frac{\Delta G}{RT}\right)}\right)\right\}} \end{array}}{4A_{0}\exp\left(-\frac{Ea}{RT}\right)\left(1-\frac{1}{\exp\left(-\frac{\Delta G}{RT}\right)}\right)}$$

*k*_1_, *k*_2_, *k*_3_, *k*_–1_, *k*_–2_, *k*_–3_ are the electron rate parameters for the forward reaction step one (eq 4), step two (eq 5) step three (eq 6), and for the backward reaction step one (eq 4), two (eq 5) and step three (eq 6). Surface coverage for hydrogen adsorption on the metal surface and the rates for hydrogen adsorption on the metal surface for reaction eq 4, eq 5, and eq 6 are presented with θ, *r*_1_, *r*_2_, and *r*_3_, respectively.

β, *F*, η, *R*, and *T* are the symmetry factor, Faraday constant, electrical overpotential, universal gas constant, and temperature, respectively.

A general diagram of the hydrogen evolution reaction (HER) in acidic and alkaline media on the catalytic surface, which is presented at eqs 1–3 and eqs 4–6, respectively, is presented in Figure 1.

## 3 Results and Discussion

### 3.1 Electrical Exchange Current for Water Electrolysis on a Fabricated Electrode with an Ir–Pt Nanoalloy

The electrical exchange current for the Tafel step in the hydrogen evolution reaction (HER) during water electrolysis on Ir–Pt nanoalloys in acidic media has been calculated. Density functional theory (DFT) calculations were extended to evaluate the exchange current density for Ir, Pt, and Ir–Pt nanoalloys with a total particle size of 30 atoms. The variation of exchange current with Ir doping in Ir–Pt nanoalloys is presented in Figure 2. The Quantum ESPRESSO package was used for DFT calculations to evaluate the exchange current of Ir–Pt nanoalloys in acidic media. In the DFT calculations, a plane wave basis set was used and the calculations were performed at the gamma point.

**Figure 2:**
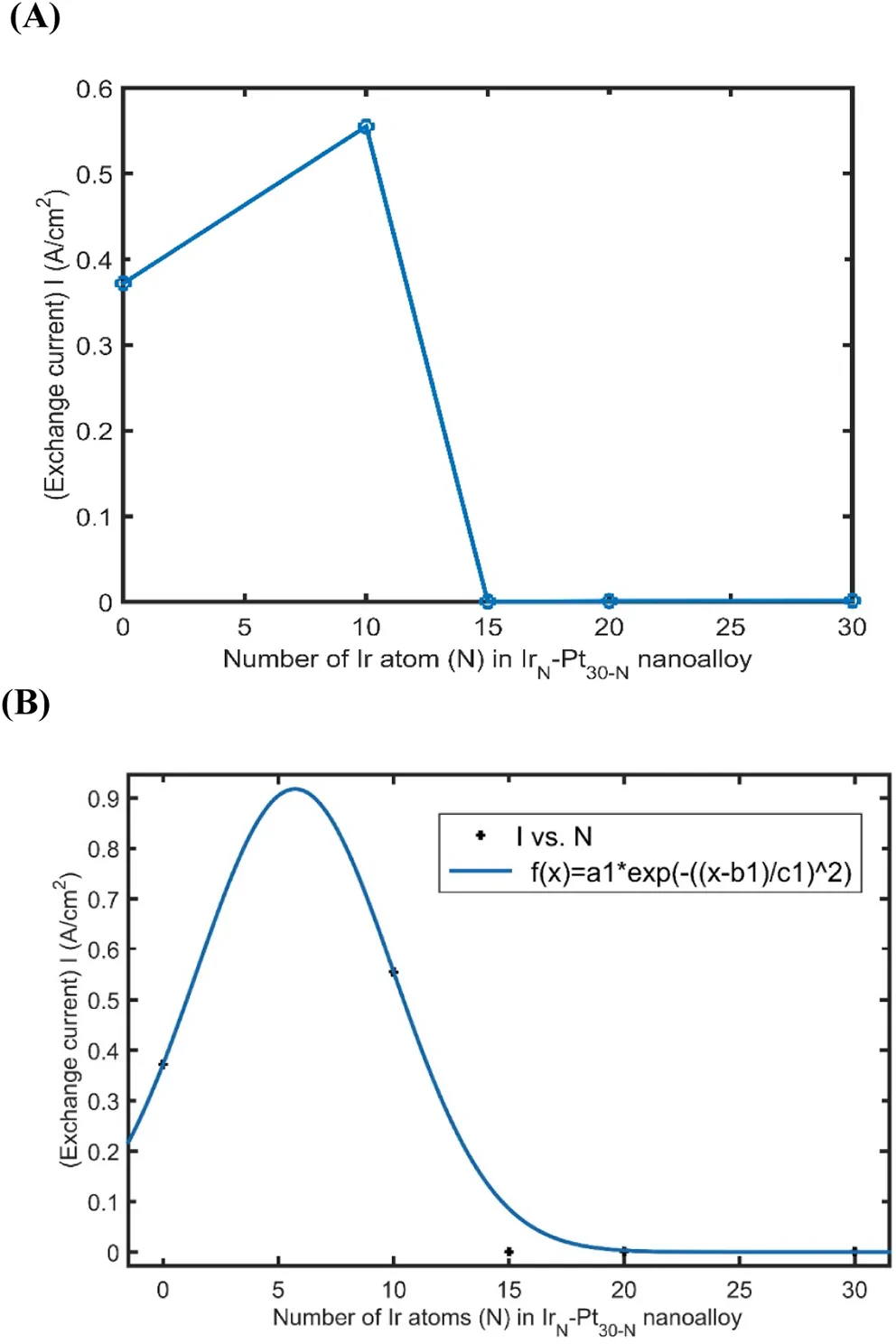
A) Exchange current for Pt–Ir nanoalloy versus Pt atoms. B) Fitted exchange current for Pt–Ir nanoalloy versus Pt atoms with a Gaussian function (blue solid line); black + symbols, real DFT calculations.

Kinetic energy cutoffs of 40 Ry for the wave functions and 400 Ry for the charge density (1 Ry = 13.606 eV) were used for the DFT calculations . In the DFT calculations, a vacuum spacing of at least 8 Å was introduced between atoms in replicated cells. Convergence thresholds of 10^–4^ Ry for energy (force) during ionic relaxation and 10^–7^ Ry for the self-consistent field (SCF) process were employed. To determine the exchange current density for the hydrogen evolution reaction (HER) on a Pt–Ir nanoalloy, DFT calculations were performed to evaluate the hydrogen adsorption energy for different Ir–Pt compositions. Using eqs 24–26, the electrical exchange current density was subsequently calculated based on eq 27.

To compute the hydrogen adsorption energy on various Ir–Pt nanoalloy compositions, the following procedure was adopted: first, the Ir–Pt nanoalloy structure was optimized; second, the hydrogen molecule was optimized; and third, hydrogen adsorption on the nanoalloy surface was optimized. The adsorption energy of hydrogen on the Ir–Pt nanoalloy was then calculated using eq 24 within the DFT framework.

According to Figure 2, in acidic media and at low Pt content, the exchange current exhibits a maximum corresponding to hydrogen adsorption on the Ir–Pt nanoalloy surface. The results indicate that, in Ir–Pt nanoalloys, hydrogen preferentially adsorbs on Ir atoms rather than on Pt atoms. Upon introducing a small amount of Ir into Pt nanoparticles, the free energy of hydrogen adsorption on the Ir sites approaches zero. Consequently, the exchange current for Ir–Pt nanoalloys is higher than that of pure Ir or pure Pt nanoparticles. DFT calculations have been used to calculate the free energy of the electrochemical reaction and the electrical potential due to ion pair.^22^

The exchange current density can be calculated from the free energy of the Tafel step in HER. $\mathrm{MH}\left(\mathrm{ads}\right)+\mathrm{MH}\left(\mathrm{ads}\right)⇌H_{2_{\left(g\right)}}+2M$ . For the calculation of free energy, the change in energy must be calculated, and it can be written as eq 24:

24 $$\Delta E=E_{(M-H)s}-(E_{(M)s}+1/2E_{H_{2}})$$

Adsorption energy at eq 24 does not explicitly account for solvation effects and surface charge compensation.

The free-energy entropy of the Tafel step reaction can be calculated using the following equation:

25 $$\Delta S_{\mathrm{Tafel step}}=\left(\Delta S_{H_{2}}+2\Delta S_{M}\right)-2\Delta S_{\mathrm{M-H}}≃\Delta S_{H_{2_{\left(g\right)}}}$$

As a result, free energy of the Tafel step reaction, ΔF, can be calculated based on eq 26:

26 $$\Delta F=\Delta E-T\Delta S_{\mathrm{Tafel step}}$$

Electrical exchange current is obtained from the free energy (eq 26):

27 $$I_{0}=k_{0}\;\exp\left(\frac{-\Delta F}{RT}\right)$$

Figure 2A also shows that the exchange current for pure Pt nanoparticles is greater than that for pure Ir nanoparticles. According to the Butler–Volmer equation, a higher exchange current leads to a higher current density than lower exchange currents. The calculated order of exchange currents, Ir–Pt > Pt > Ir, is in good agreement with available experimental data.^23,24^

The exchange current for the HER on an Ir–Pt nanoalloy is fitted by a Gaussian function, F­(x) = a_1_ exp­(−(x – b_1_)/c_1_)^2^, and gives a good fit with R-square: 0.9996 and fitting variables: a_1_ = 0.9177, b_1_ = 5.728, and c_1_ = 6.027.

The results of curve fitting by using a Gaussian function for the exchange current are presented in Figure 2B. The DFT data are shown as black points, and the Gaussian fit is shown as a solid blue line in Figure 2B.

Without loss of generality in the fundamental electrochemistry of the system, the general form of the Butler–Volmer equation is retained, and an attempt is made to express the exchange current as an exponential function of Pt doping in Ir–Pt nanoalloys for water electrolysis in acidic media.

### 3.2 Surface Coverage versus Temperature for HER

The surface coverage versus electrical overpotential at different temperatures has been considered for HER in alkaline media. Surface coverage can be presented in eq 28:

28 $$\theta =\left[-\left(k_{1}P^{-1}+k_{-1}P+k_{2}P^{-1}+k_{-2}P+2k_{-3}\right)+{\{{\left(k_{1}P^{-1}+k_{-1}P+k_{2}P^{-1}+k_{-2}P+2k_{-3}\right)}^{2}+4\left(k_{1}P^{-1}+k_{-2}P-k_{-3}\right)\left(k_{3}-k_{-3}\right)\}}^{1/2}\right]/\left\{4\left(k_{3}-k_{-3}\right)\right\}$$

Complete form of the analytical expression (eq 28) for the surface coverage is presented in the Supporting Information. In Figure 3, it is assumed that the activation energy barrier is sufficiently high, and therefore the rate constants *k*_3_ and *k*_–3_ do not change significantly with temperature. As a result, the surface coverage of metal–hydrogen as a function of electrical overpotential decreases and approaches a constant value at high electrical overpotentials. In addition, the surface coverage decreases with increasing temperature.

**Figure 3:**
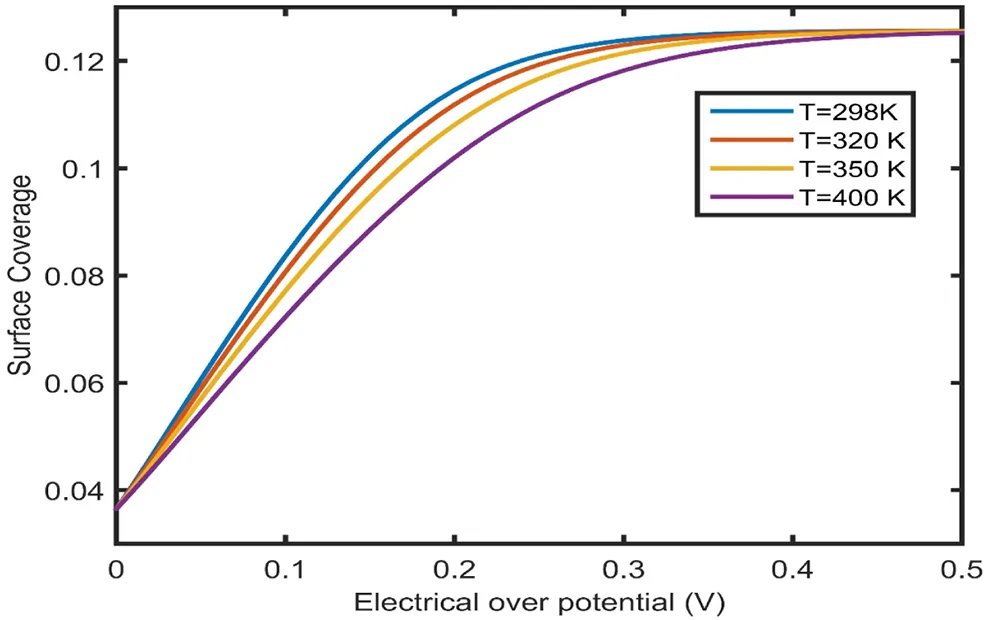
Surface coverage versus electrical overpotential at different temperatures (room temperature until 400 K).

Surface coverage has been analyzed as a function of electrical overpotential at different temperatures, and the results are presented in Figure 3. In Figure 3, eq 28 and small values of the chemical kinetics parameters *k*_1_, *k*_2_, *k*_3_, *k*_–1_, *k*_–2_, and *k*_–3_ have been used for surface coverage versus electrical overpotential in the temperature range 298–400 K.

According to Figure 3, surface coverage decreases with increasing temperature. This behavior arises because while electrostatic interactions enhance adsorption of species onto the metal surface, higher temperatures increase kinetic energy and promote desorption. Consequently, surface coverage decreases as the temperature rises.

Despite this effect, the overall trend of surface coverage versus electrical overpotential remains consistent across all temperatures. At elevated temperatures, an asymptotic limit for surface coverage is observed with respect to the electrical overpotential. This indicates that electrostatic interactions between chemical species and the metal surface contribute more strongly to adsorption than does the opposing influence of thermal kinetic energy. As a result, even at high temperatures, surface coverage ultimately approaches an asymptotic limit.

### 3.3 Estimation of the Surface Coverage of Hydrogen Adsorption in Surface Catalysis as a Function of Temperature during Water Electrolysis in Alkaline Media

There are two possible temperature ranges. For a high temperature range, the electrical overpotential is

29 $$x=\frac{\beta F\eta }{RT}\ll 1$$

Then

30 $$P=\exp\left(\frac{\beta F\eta }{RT}\right)\approx 1+\frac{\beta F\eta }{RT},P^{-1}=\exp\left(-\frac{\beta F\eta }{RT}\right)\approx 1-\frac{\beta F\eta }{RT}$$

By definition, the following variable

31 $$A=k_{1}\left(1-x\right)+k_{-1}\left(1+x\right)+k_{2}\left(1-x\right)+k_{-2}\left(1+x\right)+2k_{-3}$$

32 $$B=k_{1}\left(1-x\right)+k_{-2}\left(1+x\right)-k_{-3}$$

33 $$C=k_{3}-k_{-3}=A_{0}\exp\left(-\frac{Ea}{RT}\right)\left(1-\frac{1}{\exp\left(-\frac{\Delta G}{\mathrm{RT}}\right)}\right)$$

Surface coverage

34 $$\theta =\frac{-A+\sqrt{A^{2}+4BC}}{4C}$$

Then, surface coverage could be presented approximately as

35 $$\theta \approx \frac{\begin{array}{l} -\left[k_{1}\left(1-x\right)+k_{-1}\left(1+x\right)+k_{2}\left(1-x\right)+k_{-2}\left(1+x\right)+2k_{-3}\right]\\ +\sqrt{{[k_{1}\left(1-x\right)+k_{-1}\left(1+x\right)+k_{2}\left(1-x\right)+k_{-2}\left(1+x\right)+2k_{-3}]}^{2}+4\left\{k_{1}\left(1-x\right)+k_{-2}\left(1+x\right)-k_{-3}\right\}\left\{A_{0}\exp\left(-\frac{Ea}{RT}\right)\left(1-\frac{1}{exp\left(-\frac{\Delta G}{RT}\right)}\right)\right\}} \end{array}}{4A_{0}\exp\left(-\frac{Ea}{RT}\right)\left(1-\frac{1}{exp\left(-\frac{\Delta G}{RT}\right)}\right)}$$

Eq 35 can also be simplified for small values of the activation energy. For small activation energy

36 $$A_{0}\exp\left(-\frac{Ea}{RT}\right)\approx A_{0}\left(1-\frac{Ea}{RT}\right)$$

37 $$C=k_{3}-k_{-3}=A_{0}\exp\left(-\frac{Ea}{RT}\right)\left(1-\frac{1}{\exp\left(-\frac{\Delta G}{\mathrm{RT}}\right)}\right)\approx A_{0}\left(1-\frac{Ea}{RT}\right)\left(1-\frac{1}{\exp\left(-\frac{\Delta G}{\mathrm{RT}}\right)}\right)$$

As a result, by using eq 36 and eq 37, the surface coverage at eq 35 converts to the eq 38 approximately

38 $$\theta \approx \frac{\begin{array}{l} -\left[k_{1}\left(1-x\right)+k_{-1}\left(1+x\right)+k_{2}\left(1-x\right)+k_{-2}\left(1+x\right)+2k_{-3}\right]\\ +\sqrt{{[k_{1}\left(1-x\right)+k_{-1}\left(1+x\right)+k_{2}\left(1-x\right)+k_{-2}\left(1+x\right)+2k_{-3}]}^{2}+4\left\{k_{1}\left(1-x\right)+k_{-2}\left(1+x\right)-k_{-3}\right\}\left\{A_{0}\left(1-\frac{Ea}{RT}\right)\left(1-\frac{1}{exp\left(-\frac{\Delta G}{RT}\right)}\right)\right\}} \end{array}}{4A_{0}\left(1-\frac{Ea}{RT}\right)\left(1-\frac{1}{exp\left(-\frac{\Delta G}{RT}\right)}\right)}$$

Surface coverage could be estimated in the low-temperature range.

$P^{-1}=\exp\left(-\frac{\beta F\eta }{RT}\right)\to 0$ , eqs 18–20 reduce as following:

39 $$C=k_{3}-k_{-3}=A_{0}\exp\;\left(-\frac{Ea}{RT}\right)\left(1-\frac{1}{\exp\left(-\frac{\Delta G}{\mathrm{RT}}\right)}\right)$$

40 $$S\approx k_{-1}P+k_{-2}P+2\frac{A_{0}}{\exp\left(-\frac{\Delta G}{\mathrm{RT}}\right)}\exp\left(-\frac{Ea}{RT}\right)$$

41 $$B^{\prime }\approx k_{-2}P-\frac{A_{0}}{\exp\left(-\frac{\Delta G}{\mathrm{RT}}\right)}\exp\left(\frac{-Ea}{RT}\right)$$

Then

42 $$\theta \approx \frac{\begin{array}{l} -\left[k_{‐1}P+k_{-2}P+2\frac{A_{0}}{exp\left(-\frac{\Delta G}{RT}\right)}\exp\left(-\frac{Ea}{RT}\right)\right]\\ +\sqrt{{[k_{-1}P+k_{-2}P+2\frac{A_{0}}{exp\left(-\frac{\Delta G}{RT}\right)}\exp\left(-\frac{Ea}{RT}\right)]}^{2}+4\{k_{-2}P-\frac{A_{0}}{exp\left(-\frac{\Delta G}{RT}\right)}\exp\left(\frac{-Ea}{RT}\right)\}\left\{A_{0}\exp\left(-\frac{Ea}{RT}\right)\left(1-\frac{1}{exp\left(-\frac{\Delta G}{RT}\right)}\right)\right\}} \end{array}}{4A_{0}\exp\left(-\frac{Ea}{RT}\right)\left(1-\frac{1}{exp\left(-\frac{\Delta G}{RT}\right)}\right)}$$

### 3.4 Sensitivity of Surface Coverage to Chemical Kinetics Parameters

The sensitivity of surface coverage to kinetic parameters in alkaline media has been investigated for water electrolysis using an Ir–Pt nanoalloy with different support materials. The substrate materials can be listed based on the electrochemical reaction rate for HER as follows:

Carbon nanotube(CNT)>Ti sheet>Stainless steel

A single-walled carbon nanotube with a 15.34 Å diameter, a 40.31 Å length, and 800 carbon atoms is included as a substrate for DFT calculations. A Ti sheet is used by a basis of 24 Ti atoms, distributed in a honeycomb arrangement. The unit cell was tetragonal 7.39 × 8.53 × 20 A^3^ with periodic boundary conditions in x, y, and z in order to represent an infinitive sheet. A stainless sheet is constructed based on a Cr–Fe alloy, with a single Cr atom in a 3 × 3 × 3 supercell.^25^

The three above-mentioned substrate materials for the Ir–Pt nanoalloy induce different kinetic parameters based on the Arrhenius equation. The NEB method has been used for the calculation of quantum energy barriers and kinetic parameters for the substrate materials mentioned above.^26,27^

The Nudged Elastic Band (NEB) approach was employed to calculate the activation barriers and reaction pathways for the forward reactions in HER eqs 1 [Disp-formula eq3]. This method determines the minimum-energy path (MEP) between two local minima by generating a series of intermediate configurations (images) connected by elastic springs. The image with the highest energy is treated as the climbing image, which does not experience the spring force along the reaction path. Instead, the component of the true force along the tangent is inverted, allowing the image to move toward the first-order saddle point while minimizing the energy perpendicular to the reaction path. As a result, upon convergence, the climbing image accurately locates the transition state. The local minima identified through a preliminary structural optimization were used as the initial and final configurations for the NEB calculations, with 3–7 intermediate images generated between the endpoints.

We have extended our previous strategy^27^ for calculating the backward rate of HER on CNT, Ti sheet, and stainless-steel substrates. The backward chemical rate constant has been calculated based on the Gibbs free energy of HER in water electrolysis on different substrates as following eq 43:

43 $$K=\frac{k_{1}}{k_{-1}}=\exp\left(\frac{-\Delta G}{RT}\right)$$

where *k*_1_, *k*_–1_, and Δ*G* are the forward reaction, backward reaction, and Gibbs free energy for each step of the HER, respectively, which is shown by eqs 4–6.

Detailed kinetic parameters and Gibbs free energies of each reaction step in the HER for water electrolysis in alkaline media on different substrates are presented in Table 1.S in the Supporting Information. Surface coverage was calculated for three different values of the chemical rate constant in the first step of the electrochemical reaction, considering both the forward and backward directions. The results of surface coverage versus electrical overpotential for different kinetic parameters, for both the forward and backward reactions in the first step, are presented in Figure 4A and Figure 4B.

**Figure 4:**
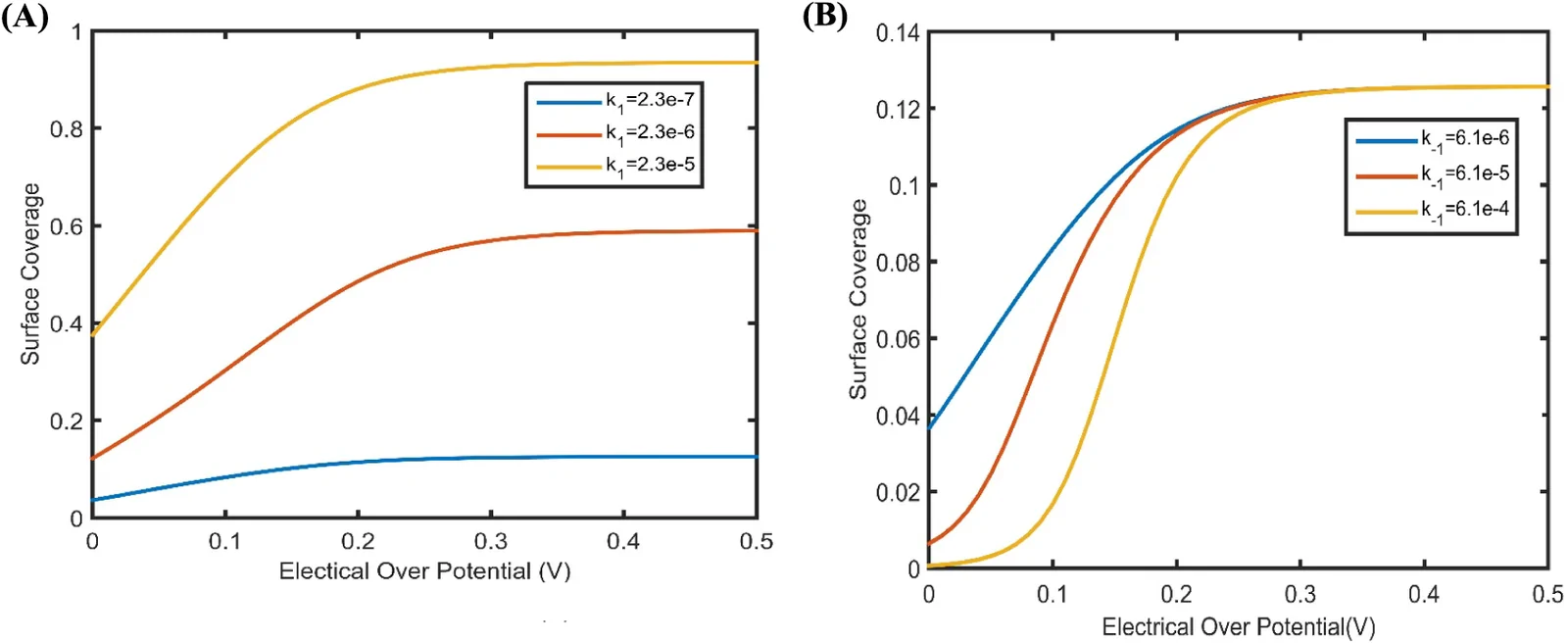
A) Surface coverage versus electrical overpotential at different chemical rate constants for the forward reaction at step 1, *k*_1_ = 2.3 × 10^–7^ (Stainless steel), 2.3 × 10^–6^ (Ti sheet), and 2.3 × 10^–5^ (CNT). B) Surface coverage versus electrical overpotential for different chemical rate constant values for the backward reaction at step 1, *k*_–1_ = 6.1 × 10^–6^ (Stainless steel), 6.1 × 10^–5^ (Ti mesh), and 6.1 × 10^–4^ (CNT).

According to Figure 4, increasing the chemical rate constant of the forward reaction in step one causes a significant change in surface coverage, which eventually approaches zero. In contrast, variations in the chemical rate constant of the backward reaction do not substantially affect the asymptotic limit or the overall surface coverage. However, at low electrical overpotentials, surface coverage is influenced by changes in the backward reaction rate constant. The HER activity for electrical overpotential versus FeO_x_(OH)_2–2x_ coverage shows nonmonotonic behavior. Nonmonotonic behavior of surface coverage depends on the order of the chemical reaction rate constant.^28^ A constant value for the surface coverage of metal–hydrogen is observed when the following condition for the reaction rate constant holds during HER.

$$K_{3}>K_{2}>K_{1}\;\mathrm{and}\;\left(K_{-1}>K_{1},K_{2}>K_{-2},K_{3}>K_{-3}\right)$$

The sensitivity of surface coverage to the chemical rate constant in the forward and backward directions of the second step in water electrolysis has been analyzed, and the results are presented in Figure 5A and Figure 5B, respectively. An asymptotic limit for surface coverage versus electrical overpotential still exists when the kinetic parameter for the backward reaction in step two (eq 2) increases. However, when the rate constant for the forward reaction in step two increases, surface coverage decreases and, at sufficiently high values of the rate constant, the surface becomes nearly empty. Furthermore, unlike the backward reaction, changing the kinetic parameter of the forward reaction in step two eliminates the asymptotic limit of surface coverage with respect to electrical overpotential.

**Figure 5:**
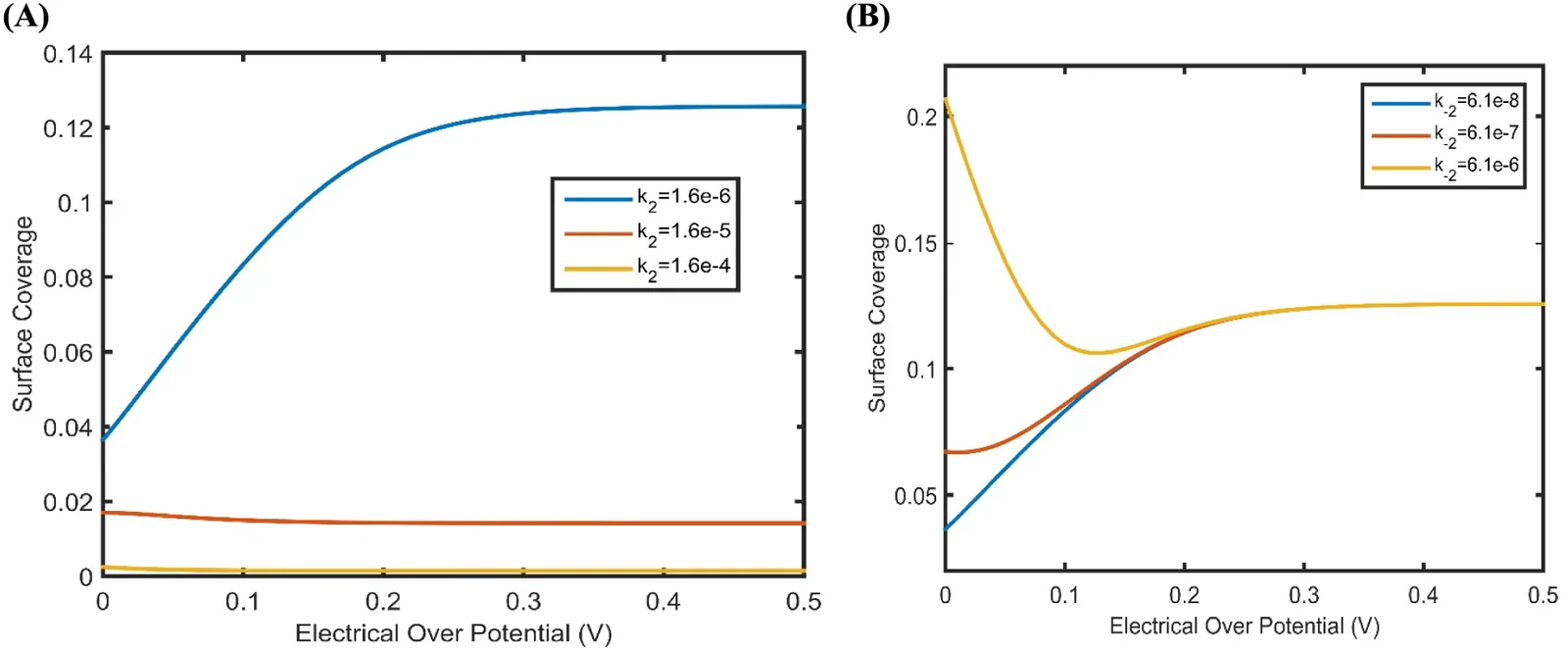
A) Surface coverage versus electrical potential at different forward reaction rate parameters for step 2, *k*_2_ = 1.6 × 10^–6^ (Stainless steel), 1.6 × 10^–5^ (Ti sheet), and 1.6 × 10^–4^ (CNT) in water electrolysis in alkaline media. B) Surface coverage versus electrical potential at different backward reaction rate parameters for step 2, *k*_*–*2_ = 6.1 × 10^–8^ (Stainless steel), 6.1 × 10^–7^ (Ti sheet), and 6.1 × 10^–6^ (CNT) in water electrolysis in alkaline media.

The sensitivity of surface coverage to the forward and backward reaction rates in step three (eq 3) of water electrolysis in alkaline media has also been investigated. The results of surface coverage sensitivity versus electrical overpotential for the forward and backward reactions are shown in Figure 6A and Figure 6B, respectively.

**Figure 6:**
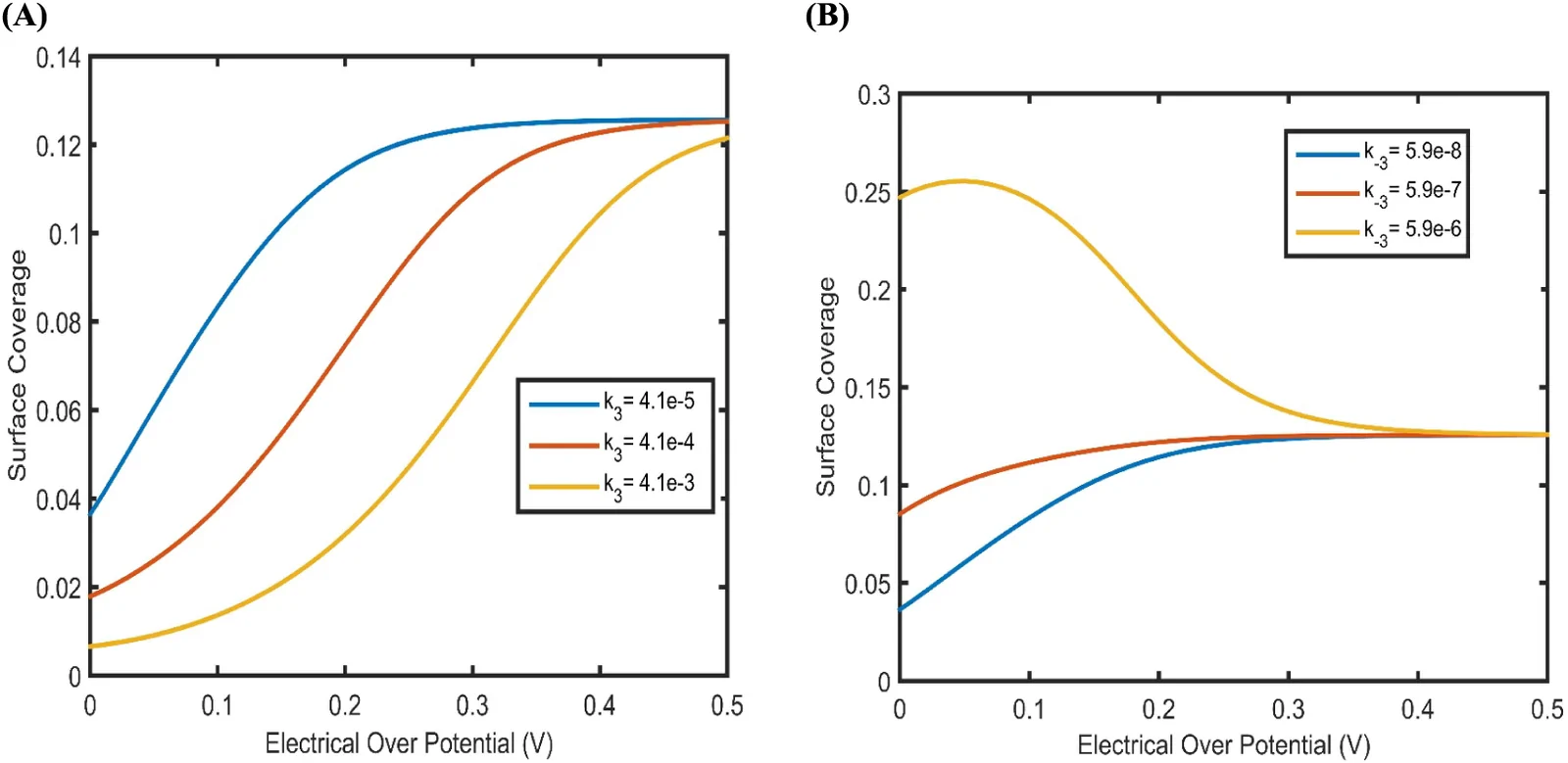
A) Surface coverage versus electrical overpotential for different values of kinetic parameters for the forward reaction at step 3 (eq 3), *k*_3_ = 4.1 × 10^–5^ (Stainless steel), 4.1 × 10^–4^ (Ti sheet), 4.1 × 10^–3^ (CNT) in water electrolysis in alkaline media. B). Surface coverage versus electrical overpotential for different values of kinetic parameters for the backward reaction at step 3 (eq 3), *k*_–3_ = 5.9 × 10^–8^ (Stainless steel), 5.9 × 10^–7^ (Ti sheet), and 5.9 × 10^–6^ (CNT) in water electrolysis in alkaline media.

According to Figure 6A, surface coverage decreases with increasing electrical overpotential when the forward reaction rate constant in step three increases, and at high overpotentials, surface coverage approaches a constant value. Similarly, Figure 6B shows that surface coverage also approaches a constant value even when the backward reaction rate constant is increased. However, in this case, increasing the backward reaction rate constant leads to higher surface coverage, and an asymptotic limit for surface coverage versus electrical overpotential is observed.

This behavior is a general result for the surface coverage of metal–hydrogen as a function of electrical overpotential when the chemical reaction rate constants are of this order of magnitude. Any substrate that influences the chemical rate constants on the Ir–Pt nanocatalyst is expected to exhibit a similar dependence of metal–hydrogen surface coverage on electrical overpotential

## 4 Conclusion

Water electrolysis has been investigated on Ir–Pt nanoalloys (total size of 30 atoms) for the hydrogen evolution reaction (HER) in acidic media. The results show that the exchange current exhibits a maximum at low Ir content in Ir–Pt nanoalloys during HER which confirm with the available experimental data. Furthermore, the free energy of the Tafel step on Ir–Pt nanoalloy electrodes with a small amount of Ir in Pt deviates only slightly from zero during hydrogen adsorption in acidic media. DFT calculations show an excellent exponential dependence of the exchange current density on Pt content in Pt–Ir nanoalloys for water electrolysis in acidic media. Although the exchange current density of Ir_1–x_–Pt_x_ exhibits a complex dependence on composition (x) or Pt content, the general form of the Butler–Volmer equation is retained for describing the electrical current density of the electrochemical system using Ir–Pt nanoalloy electrodes in acidic media. The exchange current for Pt nanoparticles is higher than that for Ir nanoparticles of the same size (30 atoms). A novel analytical expression has been presented for surface coverage over both high- and low-temperature ranges, as a function of electrical overpotential, Gibbs free energy of the Tafel step, activation energy of the Tafel step, and chemical reaction rates not involved in the Tafel step of the hydrogen evolution reaction (HER) during water electrolysis in alkaline media. Surface coverage versus electrical overpotential in alkaline media for Ir–Pt nanoalloys was analyzed across a temperature range of 298 K ≤ *T* ≤ 400 K. The results indicate that surface coverage decreases at higher temperatures due to enhanced desorption, but an asymptotic limit is observed, as electrostatic interactions dominate over thermal effects. Notably, variations in temperature do not alter the overall trend of surface coverage versus electrical overpotential in alkaline conditions. The sensitivity of surface coverage to electrical overpotential for the Ir–Pt nanoalloy which is supported on different substrate materials has also been evaluated for all steps of the electrochemical reaction in alkaline media. The results demonstrate that surface coverage is particularly sensitive to the forward reaction rate constant in the first step of water electrolysis. Convergence to an identical value of surface coverage versus electrical overpotential for the Ir–Pt nanoalloy, including substrate effects for water electrolysis in alkaline media, depends mostly on the backward reaction steps.

## Supplementary Material



## Notes

Analytical expression for surface coverage of hydrogen–metal catalysts and kinetic parameters for HER on different substrates (PDF)
