# Supplementary material for: A Theoretical Approach to the Effect of Substrate Engineering on Hydrogen–Metal Surface Coverage in Ir–Pt Nanocatalysts for Water Electrolysis
Source: ACS Omega. 2026 Aug 21;11(35):53176–85. doi: 10.1021/acsomega.6c05827 (PMC13563992; doi:10.1021/acsomega.6c05827)
Supplement: Supplementary file 1 [file ao6c05827_si_001.pdf]

Theoretical Approach for Substrate Engineering Effect on Hydrogen-Metal Surface Coverage in Ir–Pt Nanocatalysts in Water Electrolysis

Farid Taherkhani[1,2]*, Fabian Mauss[1,2]

[1]*Departments of Thermodynamics and Thermal Process Engineering Brandenburg University of Technology, Cottbus, 03046, Germany*

[2] *Energiespeicher-und Energiewandlersysteme, Brandenburgische Technische Universität Cottbus–Senftenberg, Germany*

Corresponding Authors: taherkha@b-tu.de, faridtaherkhani@gmail.com

1. Analytical expression for surface coverage of hydrogen-metal catalyst

Analytical expression for surface coverage of hydrogen adsorption on metal surface as a function of electrical over potential and temperature for HER in water electrolysis in alkaline media

$$\mathrm{M+H2O}+e\rightleftharpoons \mathrm{MH(ad)+OH^-} \quad (1)$$
$$\mathrm{MH(ad)+H2O}+e\rightleftharpoons \mathrm{H2+M+OH^-} \quad (2)$$
$$\mathrm{MH(ads)+MH(ads)}\rightleftharpoons \mathrm{H_2+2M} \quad (3)$$

has been presented as a following

$$v_1 = k_1(1-\theta)P^{-1} - k_{-1}\theta P \quad (4)$$
$$v_2 = k_2\theta P^{-1} - k_{-2}\theta(1-\theta)P \quad (5)$$
$$v_3 = k_3\theta^2 - k_{-3}(1-\theta)^2 \quad (6)$$

Where P is defined

$$P = \exp(\frac{\beta F \eta}{RT}) \qquad (7)$$

Making stationary assumption

$$v_1 - v_2 - 2\ v_3 = 0 \qquad (8)$$

As a result, surface coverage could be obtained as a following equation analytically

θ= -(exp(-(B*F*V)./(R*T)).*(k1 + k2 - (k1^2 + 2*k1*k2 + 2*exp((2*B*F*V)./(R*T))*k1*k1a + 2*exp((2*B*F*V)./(R*T))*k1*k2a + 8*k3*exp((B*F*V)./(R*T))*k1 + k2^2 + 2*exp((2*B*F*V)./(R*T))*k2*k1a + 2*exp((2*B*F*V)./(R*T))*k2*k2a + 8*k3a*exp((B*F*V)./(R*T))*k2 + exp((4*B*F*V)./(R*T))*k1a^2 + 2*exp((4*B*F*V)./(R*T))*k1a*k2a + 8*k3a*exp((3*B*F*V)./(R*T))*k1a + exp((4*B*F*V)./(R*T))*k2a^2 + 8*k3*exp((3*B*F*V)./(R*T))*k2a + 16*k3*k3a*exp((2*B*F*V)./(R*T))).^(1/2) + k1a*exp((2*B*F*V)./(R*T)) + 4*k3a*exp((B*F*V)./(R*T)) + k2a*exp((2*B*F*V)./(R*T)))))./(4*(k3 - k3a)); (9)

2. Kinetics Parameter for HER in different substrates

Free energy and backward reaction for HER in alkaline media for reactions which are described by Eqs 1-3 for stainless steel substrate, Ti sheet, carbon nanotube (CNT)

Table.1.S

Free Energy (J/mol)	$8.12*10^{3}$	$-8.09*10^{3}$	$-1.62*10^{4}$
Backward kinetics Parameter	$6.1*10^{-6}$	$6.1*10^{-8}$	$5.9*10^{-8}$
Reaction number for substrate (Stainless steel)	1	2	3

Free Energy (J/mol)	$8.12*10^{3}$	$-8.09*10^{3}$	$-1.62*10^{4}$
Backward kinetics Parameter	$6.1*10^{-5}$	$6.1*10^{-7}$	$5.9*10^{-7}$
Reaction number for substrate (Ti sheet)	1	2	3

Free Energy (J/mol)	$8.12*10^{3}$	$-8.09*10^{3}$	$-1.62*10^{4}$
Backward kinetics Parameter	$6.1*10^{-4}$	$6.1*10^{-6}$	$5.9*10^{-6}$
Reaction number for substrate (CNT)	1	2	3

For forward reaction 1-3 following rate constant is obtained.

Chemical kinetics parameter of HER for Reaction number based on substrate Stainless steel for forward reaction at Eq1-3 on Pt-Ir nanoalloy

k1=2.3e-7; k2=1.6e-6; k3=4.1e-5;

Also, chemical kinetics parameter of HER for Reaction number based on substrate Ti sheet for Ir-Pt nanoalloy

k1=2.3e-6; k2=1.6e-5; k3=4.1e-4;

Also, chemical kinetics parameter of HER for Reaction number based on substrate CNT for Ir-Pt nanoalloy

k1=2.3e-5; k2=1.6e-4; k3=4.1e-3;
